# GSK-3*β* Inhibition Attenuates CLP-Induced Liver Injury by Reducing Inflammation and Hepatic Cell Apoptosis

**DOI:** 10.1155/2014/629507

**Published:** 2014-11-30

**Authors:** Hui Zhang, Wenjie Wang, Haoshu Fang, Yan Yang, Xiaolan Li, Junli He, Xiaojing Jiang, Weipeng Wang, Shenpei Liu, Jifa Hu, Anding Liu, Uta Dahmen, Olaf Dirsch

**Affiliations:** ^1^Department of Clinical Laboratory, Hubei Maternal and Child Health Hospital, Wuhan 430070, China; ^2^Department of Infectious Diseases, Wuhan General Hospital of Guangzhou Military Command, Wuhan 430070, China; ^3^Department of Pathophysiology, Anhui Medical University, Hefei 230032, China; ^4^Experimental Medicine Center, Tongji Hospital, Tongji Medical College, Huazhong University of Science and Technology, No. 1095 Jiefang Avenue, Wuhan 430030, China; ^5^Department of Molecular Medical Center, Tongji Hospital, Tongji Medical College, Huazhong University of Science and Technology, Wuhan 430030, China; ^6^Experimental Transplantation Surgery, Department of General, Visceral and Vascular Surgery, University Hospital of Jena, Friedrich-Schiller-University Jena, 07747 Jena, Germany

## Abstract

Liver dysfunction has been known to occur frequently in cases of sepsis. Excessive inflammation and apoptosis are pathological features of acute liver failure. Recent studies suggest that activation of glycogen synthase kinase- (GSK-) 3*β* is involved in inflammation and apoptosis. We aimed to investigate the protective effects of GSK-3*β* inhibition on polymicrobial sepsis-induced liver injury and to explore the possible mechanisms. Polymicrobial sepsis was induced by cecal ligation and puncture (CLP), and SB216763 was used to inhibit GSK-3*β* in C57BL/6 mice. GSK-3*β* was activated following CLP. Administration of SB216763 decreased mortality, ameliorated liver injury, and reduced hepatic apoptosis. The inhibition of GSK-3*β* also reduced leukocyte infiltration and hepatic inflammatory cytokine expression and release. Moreover, GSK-3*β* inhibition suppressed the transcriptional activity of nuclear factor-kappa B (NF-*κ*B) but enhanced the transcriptional activity of cAMP response element binding protein (CREB) in the liver. In in vitro studies, GSK-3*β* inhibition reduced inflammatory cytokine production via modulation of NF-*κ*B and CREB signaling pathways in lipopolysaccharide-stimulated macrophages. In conclusion, these findings suggest that GSK-3*β* blockade protects against CLP-induced liver via inhibition of inflammation by modulating NF-*κ*B and CREB activity and suppression of hepatic apoptosis.

## 1. Introduction

Sepsis is defined as the acute systemic inflammatory response to infection with a clinical spectrum ranging from hemodynamic changes to multiple organ dysfunction syndrome and even death [1–3]. It is estimated that there are 750,000 cases of sepsis with a mortality rate between 30% and 50% in North America per year [4]. Liver dysfunction has been known to occur frequently in cases of sepsis. The incidences of sepsis-associated liver dysfunction and liver failure range from 34% to 46% and from 1.3% to 22% in patients with sepsis, respectively [5]. Attenuating liver injury and restoring liver function lower morbidity and mortality rates in patients with sepsis [5].

The mechanisms involved in sepsis-induced liver injury are diverse, including uncontrolled systemic inflammatory activation, hepatic ischemia, coagulopathy, and deregulated cell apoptosis [6–9]. In sepsis, the liver plays a central role in bacterial phagocytosis and clearance. Meanwhile, an inflammatory response is triggered during bacterial clearance in sepsis. Inflammatory mediators, such as tumor necrosis factor- (TNF-) *α*, interleukin- (IL-) 1, IL-6, interferon- (IFN-) *γ*, IL-8, nitric oxide (NO), and reactive oxygen species (ROS), are released from liver resident and infiltrated immune cells, which mediate sepsis-induced liver injury [5]. Apoptotic cell death has a prominent role in the evolution of organ damage following sepsis. Animal studies have demonstrated that blocking apoptosis can improve outcome in experimental models of severe sepsis [9–11].

Glycogen synthase kinase- (GSK-) 3*β*, a ubiquitously expressed serine/threonine protein kinase, is initially identified as a regulator of glycogen metabolism [12, 13]. Beyond its role in glycogen metabolism, recent studies have implicated that GSK-3*β* plays an important role in regulation of inflammatory response [14–16]. GSK-3*β* inhibition reduced the production of TNF-*α* and IL-6 and enhanced IL-10 production in lipopolysaccharide- (LPS-) stimulated monocytes [14, 15]. Furthermore, GSK-3*β* inhibition effectively protected mice and rats from endotoxin shock [15, 16] or mice from live bacterial infection [17, 18]. Additionally, recent studies have demonstrated that inhibition of GSK-3*β* activity prevents apoptotic cell death. Mice treated with GSK-3*β* inhibitors showed decreased renal cell apoptosis in response to endotoxemia [19]. Moreover, GSK-3*β* blockade significantly reduced hepatic apoptotic cell death in response to D-galactosamine/LPS-induced liver injury [20] or ischemia/reperfusion (I/R) injury [21, 22], respectively. Although the role of GSK-3*β* in endotoxin shock and live bacterial infection has been extensively investigated, less information is available on the possible effects of GSK-3*β* inhibition in polymicrobial sepsis, which is more representative of the clinical condition.

Therefore, we aimed to investigate the protective effects of GSK-3*β* inhibition on polymicrobial sepsis-induced liver injury by a mouse cecal ligation and puncture (CLP) model and to further explore the possible mechanisms.

## 2. Materials and Methods

### 2.1. Animals

Male inbred C57BL/6 mice (8–10 weeks old, weighing within 20~22 g) were purchased from Wuhan University Center for Animal Experiment (Wuhan, China). All animals were housed under standard animal care conditions and had free access to water and food. All procedures were carried out according to the ethical guidelines of the Animal Care and Use Committee of Huazhong University of Science and Technology.

### 2.2. CLP Model of Sepsis

Sepsis was induced in mice using the CLP method. Mice were completely anesthetized with pentobarbital (60 mg/kg, i.p.) and a midline abdominal incision was performed. After exposing the cecum and manipulating the stool to the tip of the cecum, the cecum was ligated 1 cm from the tip and was perforated by one through-and-through puncture (two holes) with a 20-gauge needle to induce polymicrobial peritonitis. The abdominal wall was closed in two layers. Sham-operated animals underwent laparotomy and bowel manipulation without ligation and perforation. All mice had free access to food and water after recovery from anesthesia. To investigate the role of GSK-3*β* in CLP-induced liver injury, mice were treated with SB216763 (25 mg/kg, i.p. Sigma-Aldrich, St. Louis, MO) or vehicle (10% dimethyl sulfoxide, DMSO, Sigma-Aldrich) at 1 h, 6 h, and 12 h following CLP. The dose of SB216763 treatment was selected following previous studies [22, 23]. Mice were killed at 6 h and 20 h of reperfusion. Liver injury, hepatic apoptosis, inflammatory cytokines, transcription factor nuclear factor-*κ*B (NF-*κ*B) activation, and cAMP response element binding protein (CREB) activation were analyzed.

### 2.3. Liver Damage Assessment

To assess hepatocellular injury following CLP, serum alanine transaminase (ALT) and aspartate aminotransferase (AST) were measured using an Automated Chemical Analyzer (Hitachi Co., Tokyo, Japan).

### 2.4. Histopathology

Liver tissue was fixed in 4.5% buffered formalin for at least 24 h. Paraffin embedding was performed using standard techniques. Sections (4 *μ*m) were stained with hematoxylin-eosin.

### 2.5. MPO Immunohistochemistry

After deparaffinization, rehydration, and antigen retrieval, sections were incubated with myeloperoxidase (MPO) antibody (1 : 50; Abcam, Cambridge, UK) for 1 h at room temperature, followed by incubation with goat anti-rabbit secondary antibody, and then visualized with a diaminobenzidine kit. MPO-positive cells were counted in 5 high-power fields (HPFs)/section under a microscope (×400), and the number of cells/field was shown.

### 2.6. RAW264.7 Cell Culture

The murine macrophage cell line RAW264.7 was purchased from the Type Culture Collection of the Chinese Academy of Sciences (Shanghai, China). Cells were cultured in DMEM medium (Invitrogen, Carlsbad, CA) supplemented with 10% fetal calf serum, 2 mM glutamine, penicillin (100 IU/mL), and streptomycin (100 *μ*g/mL) at 37°C under a gas phase of air/CO_2_ (95 : 5). Cells were plated at a density of 5 × 10^5^ cells/well in a 24-well plate for cytokine assay or 2 × 10^6^ cells/well in a 6-well plate for western blotting. LPS (10 ng/mL) was used to activate cells. SB216763 (10 *μ*M) was used to inhibit GSK-3*β*.

### 2.7. Caspase-3 Activity Assay

Relative caspase-3 activity in livers was detected with a caspase-3 colorimetric assay kit (Abcam) according to the manufacturer's instructions.

### 2.8. NF-*κ*B and CREB Activity Assay

Nuclear and cytoplasmic extraction kit was bought from Pierce (Thermo Pierce, Rockford, IL). Nuclear proteins from liver tissues or RAW264.7 cells were isolated according to the instructions of the manufacturer. Levels of NF-*κ*B p65 and CREB activity in the nuclear extracts were quantified by TransAM NF-*κ*B p65 and TransAM CREB assay kits (Active Motif, Carlsbad, CA) according to the manufacturer's instructions.

### 2.9. Enzyme-Linked Immunosorbent Assay

The levels of TNF-*α* and IL-6 in serum and cultured medium were analyzed using commercially available enzyme-linked immunosorbent assay (ELISA) kits (R&D Systems, Minneapolis, MN).

### 2.10. Gel Electrophoresis and Western Blotting

Detection of the proteins by western blotting was performed as described previously [21]. Equivalent amounts of protein were separated on 12% gels by sodium dodecyl sulfate-polyacrylamide gel electrophoresis and transferred to polyvinyldifluoride membranes (GE Healthcare, Buckinghamshire, UK). After blocking, the membranes were incubated with primary antibodies: rabbit anti-phospho-GSK-3*β* (Ser9) (1 : 1000; Cell Signaling Technology, Beverly, MA), rabbit anti-GSK-3*β* (1 : 1000, Cell Signaling Technology), rabbit anti-phospho-glycogen synthase (1 : 1000, Cell Signaling Technology), rabbit anti-cleaved caspase-3 (1 : 1000, Cell Signaling Technology), rabbit anti-cleaved caspase-7 (1 : 1000, Cell Signaling Technology), and anti-glyceraldehyde-3-phosphate dehydrogenase (GAPDH) (1 : 20000; Sigma-Aldrich), at 4°C with gentle shaking overnight. Detection was carried out using horseradish peroxidase-conjugated goat anti-rabbit IgG antibody (1 : 3000, Abcam), followed by ECL western blotting detection reagents (GE Healthcare, Buckinghamshire, UK). Western blots were visualized on the Kodak Image Station (Carestream Health Inc., Rochester, NY).

### 2.11. Quantitative Polymerase Chain Reaction

Real time polymerase chain reaction (PCR) was performed as described previously [24]. Total RNA was isolated by TRIzol Reagent (Invitrogen) according to the manufacturer's instruction. cDNA synthesis was performed using the First-Strand cDNA synthesis kit (Invitrogen). Quantitative PCR was performed using a Roche Light cycle system (Roche, Rotkreuz, Switzerland) with SYBR green master mix (Qiagen, Hilden, Germany) and primers. Amplification conditions were 95°C (10 min) followed by 50 cycles of 95°C (30 s) and 60°C (20 s). Primers used to amplify a specific mouse gene fragments were as follows: TNF-*α* forward: 5′-TGCTGGGAAGCCTAAAAGG-3′; reverse: 5′-CGAATTTTGAGAAGATGATCCTG-3′, IL-6 forward: 5′-TCAATTCCAGAAACCGCTATGA-3′; reverse: 5′-CACCAGCATCAGTCCCAAGA-3, IL-1*β* forward: 5′-CAGGTCGCTCAGGGTCACA-3′; reverse: 5′-CAGAGGCAAGGAGGAAACACA-3, IL-10 forward: 5′-CACAAAGCAGCCTTGCAGAA-3′; reverse: 5′-AGAGCAGGCAGCATAGCAGTG-3, and *β*-actin forward: 5′-AGAGGGAAATCGTGCGTGAC-3′; reverse: 5′-CAATAGTGATGACCTGGCCGT-3′. Relative quantification of target mRNA expression was calculated and further normalized to housekeeping gene *β*-actin.

### 2.12. Statistical Analysis

The data are expressed as means ± SD. Differences between groups were evaluated for significance by one way ANOVA analysis combined with Bonferroni post hoc test (comparison type: versus vehicle-treated group). The Kaplan-Meier survival analyses were performed using a log rank test. All tests were performed using SigmaStat v3.5 (Systat-Software, Erkrath, Germany). A *P* value below 0.05 was considered statistically significant.

## 3. Results

### 3.1. Inhibition of GSK-3*β* Ameliorates CLP-Induced Liver Injury and Improves the Survival

**Figure 1:**
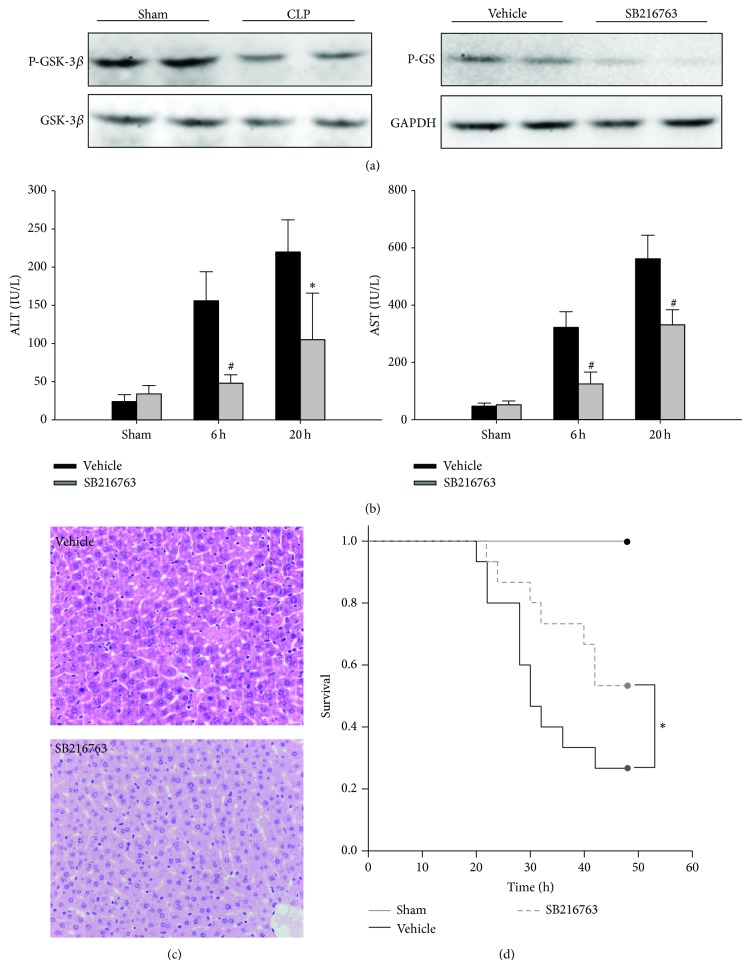
GSK-3*β* inhibition improves survival and protects against CLP-induced liver injury. The septic mice were treated with SB216763 (25 mg/kg, i.p.) or vehicle (DMSO) at 1 h, 6 h, and 12 h after CLP. (a) Liver samples were harvested at 6 h after CLP and were subjected to western blotting analysis of phosphorylated (serine 9) GSK-3*β* and phosphorylated glycogen synthases (GS). (b) ALT and AST levels were analyzed as a measure of hepatocellular injury. Data are shown as mean ± SD. ^*^ *P* < 0.05, ^#^ *P* < 0.01 compared with vehicle-treated group. (c) The liver specimens were sampled at 20 h after CLP and stained with hematoxylin and eosin staining (original magnification ×400). Representative images from 6 mice/group were selected. (d) The Kaplan-Meier method was used to determine the difference of survival rate after CLP. *P* value was analyzed by log rank test. ^*^ *P* < 0.05 compared with vehicle-treated group.

To determine whether GSK-3*β* inhibition could attenuate liver injury during sepsis, GSK-3*β* inhibitor SB216763 was administrated to mice at 1 h, 6 h, and 12 h following CLP. As shown in Figure 1(a), compared with sham controls, the phosphorylated (serine 9) GSK-3*β* levels in the liver tissue were reduced at 6 h following CLP, suggesting GSK-3*β* was activated by the CLP insult. The inhibition of liver GSK-3*β* activity in vivo was indicated by the reduced phosphorylation of glycogen synthase, a downstream substrate of GSK-3*β*. As shown in Figure 1(b), compared with the vehicle-treated group, CLP resulted in significant increase in the serum levels of ALT and AST. However, SB216763 administration significantly attenuated the liver injury caused by CLP. The histologic examination showed significant liver tissue injury following CLP, as indicated by swollen hepatocytes and leukocyte infiltration. The inhibition of GSK-3*β* resulted in significant attenuation of these changes (Figure 1(c)). To address whether GSK-3*β* inhibition was of benefit for septic mice induced by CLP, survival was then assessed at 48 h following CLP. As shown in Figure 1(d), GSK-3*β* inhibitor SB216763 treatment significantly increased the survival rate of mice. Vehicle-treated mice had a 48 h survival of 27%. In contrast, the survival rate of mice treated with SB216763 was 53%.

### 3.2. Inhibition of GSK-3*β* Modulates the Proinflammatory Cytokine Expression and the Neutrophil Infiltration following CLP

**Figure 2:**
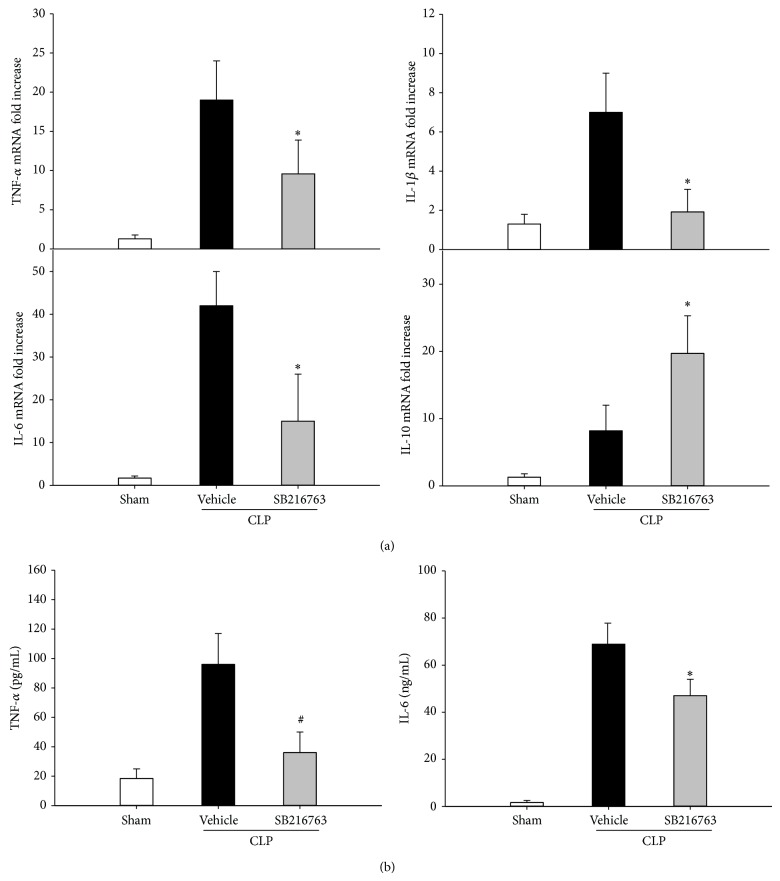
GSK-3*β* inhibition attenuates the inflammatory cytokine production following CLP. (a) GSK-3*β* inhibition altered mRNA expression of TNF-*α*, IL-6, IL-1*β*, and IL-10 in the liver of septic mice. (b) Serum TNF-*α* and IL-6 levels were assessed by ELISA. Data are shown as mean ± SD. *n* = 6 per group. ^*^ *P* < 0.05, ^#^ *P* < 0.01 compared with vehicle-treated group.

**Figure 3:**
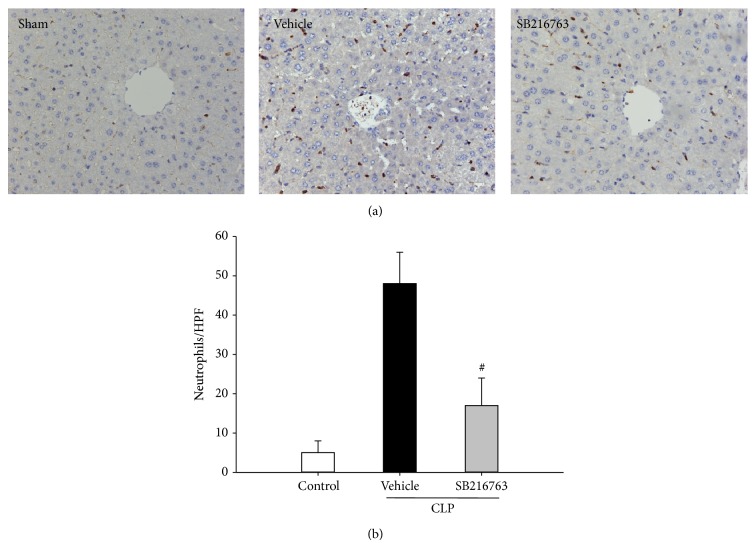
GSK-3*β* inhibition reduces the neutrophil infiltration in liver following CLP. (a) MPO staining was performed on liver sections from mice at 20 h after CLP. (b) The numbers of MPO-positive neutrophils that infiltrated the liver were determined (original magnification ×400). Representative images from 6 mice/group were selected. Data are shown as mean ± SD. *n* = 6 per group. ^#^ *P* < 0.01 compared with vehicle-treated group.

To test whether GSK-3*β* inhibition could attenuate liver injury during sepsis via inhibition of inflammation, the production of TNF-*α*, IL-6, IL-1*β*, and IL-10 was analyzed at 6 h following CLP. As shown in Figure 2(a), CLP resulted in significant increase in the mRNA levels of TNF-*α*, IL-6, IL-1*β*, and IL-10. Liver TNF-*α*, IL-6, and IL-1*β* mRNA levels were significantly attenuated by the administration of SB216763. In contrast, the IL-10 mRNA levels were significantly increased by the SB216763 administration. Furthermore, the inhibition of GSK-3*β* significantly reduced the CLP-induced production of serum TNF-*α* and IL-6 levels (Figure 2(b)). The numbers of MPO staining positive neutrophils that infiltrated the liver were determined. Hepatic neutrophil infiltration was significantly increased at 20 h following CLP. However, SB216763 administration significantly reduced the CLP-induced increase in the infiltration of neutrophils (Figure 3). These results indicated that inhibition of GSK-3*β* relieved the inflammation in the liver following CLP.

### 3.3. Inhibition of GSK-3*β* Modulates Transcription Factor Activities following CLP

**Figure 4:**
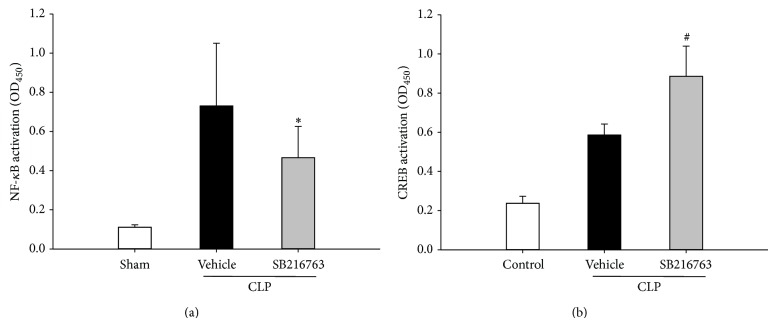
GSK-3*β* inhibition modulates the NF-*κ*B and CREB activation following CLP. Mice were subjected to CLP and treated with either SB216763 (25 mg/kg, i.p.) or vehicle (DMSO). Liver samples were harvested at 20 h after CLP. NF-*κ*B activity, as well as CREB activity, was assessed. Data are shown as mean ± SD. *n* = 6 per group. ^*^ *P* < 0.05, ^#^ *P* < 0.01 compared with vehicle-treated group.

To obtain insight into the underlying mechanisms responsible for the anti-inflammatory effect of GSK-3*β* inhibition, the effect of GSK-3*β* inhibition on NF-*κ*B and CREB activity was assessed. The activity of NF-*κ*B and CREB was significantly increased following CLP. However, NF-*κ*B activity was significantly inhibited in SB216763-treated animals in response to CLP insult compared to vehicle-injected animals. Conversely, activated nuclear CREB was enhanced in the liver after the inhibition of GSK-3*β* (Figure 4).

### 3.4. Inhibition of GSK-3*β* Modulates Transcription Factor Activities in Macrophages

**Figure 5:**
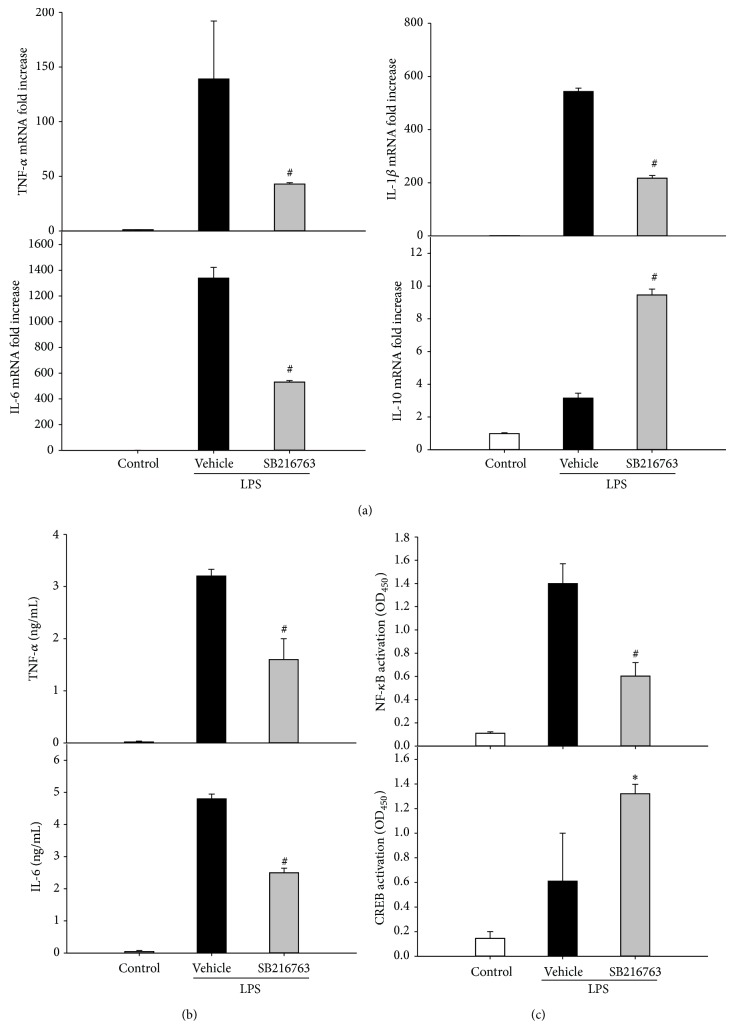
GSK-3*β* inhibition reduces the proinflammatory cytokine expression in cultured macrophages stimulated by LPS. (a) RAW264.7 cells were stimulated with LPS for 6 h in the absence or presence of SB216763 (10 *μ*M). TNF-*α*, IL-6, IL-1*β*, and IL-10 mRNA expression levels were measured by quantitative PCR. (b) TNF-*α* and IL-6 concentration in the cultured medium was measured by ELISA. (c) RAW264.7 cells were stimulated with LPS for 1 h in the absence or presence of SB216763 (10 *μ*M). NF-*κ*B activity, as well as CREB activity, was assessed. Data are shown as mean ± SD. *n* = 5 per group. ^*^ *P* < 0.05, ^#^ *P* < 0.01 compared with vehicle-treated group.

To further gain insight into the underlying signal transduction pathway involved in the regulation of inflammatory response in CLP by GSK-3*β*, the effect of GSK-3*β* inhibition on the production of proinflammatory cytokines in macrophages was assessed in vitro. RAW264.7 cells were stimulated with LPS in the absence or presence of SB216763. As shown in Figure 5(a), the GSK-3*β* inhibition significantly reduced the production of TNF-*α*, IL-6, and IL-1*β* but increased IL-10 gene induction at 6 h of culture. Similarly, TNF-*α* and IL-6 protein levels in the cultured medium were increased dramatically after treatment with LPS for 6 h; GSK-3*β* inhibition reduced the production of TNF-*α* and IL-6 levels. To investigate whether the anti-inflammatory effect of GSK-3*β* inhibition was mediated via NF-*κ*B and CREB pathways, we examined the effect of GSK-3*β* inhibition on the activity of nuclear NF-*κ*B and CREB. As shown in Figure 5, GSK-3*β* inhibition significantly inhibited NF-*κ*B activity but increased CREB activity.

### 3.5. Inhibition of GSK-3*β* Decreases Hepatic Apoptosis

**Figure 6:**
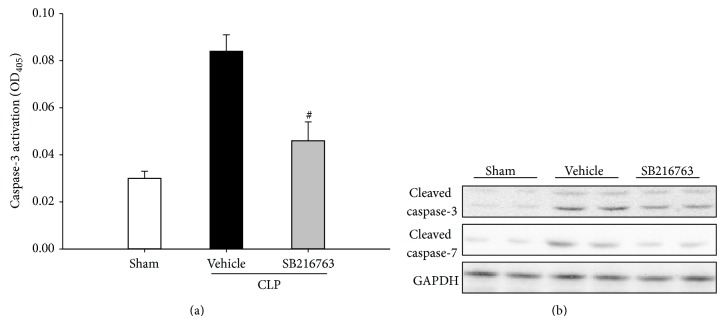
GSK-3*β* inhibition prevents the CLP-induced hepatic apoptosis. (a) Mice were subjected to CLP and treated with either SB216763 (25 mg/kg, i.p.) or vehicle (DMSO). Liver samples were harvested at 20 h after CLP. The caspase-3 activity was measured. Data are shown as mean ± SD. *n* = 6 per group. ^#^ *P* < 0.01 compared with vehicle-treated group. (b) The expression levels of the cleaved caspase-3 and the cleaved caspase-7 were examined by western blotting analysis.

To test whether GSK-3*β* inhibition could attenuate hepatic apoptosis, the caspase-3 activity in the liver was evaluated at 20 h following CLP insult. Compared to the activity in the sham operated animals, the caspase-3 activity was significantly increased in the vehicle-treated animals. The inhibition of GSK-3*β* significantly reduced the induction of caspase-3 activity by CLP (Figure 6(a)). Furthermore, the vehicle-treated animals expressed higher levels of cleaved caspase-3 and cleaved caspase-7 protein, which were significantly reduced by the GSK-3*β* inhibition (Figure 6(b)). This finding indicated that inhibition of GSK-3*β* could attenuate CLP-induced apoptosis.

## 4. Discussion

Recent studies suggest that GSK-3*β* inhibition has protective properties in LPS-induced septic shock. However, the role of GSK-3*β* inhibition in the pathogenesis of live injury induced by polymicrobial sepsis and its regulatory mechanisms remain poorly understood. In the present study, we documented that GSK-3*β* inhibition protected against CLP-induced liver injury via inhibition inflammatory response and hepatic apoptosis. Our data also suggested that NF-*κ*B and CREB mediated mechanisms are the prominent pathways involved in the protective effects of GSK-3*β* inhibition in the CLP-induced liver injury.

Inhibition of GSK-3*β* suppresses systemic inflammation and confers protection against CLP-induced liver injury. GSK-3*β* is involved in many intracellular functions including regulating cell activation, differentiation, and survival [12, 13]. Among the diverse functions that are regulated by GSK-3*β*, inflammation has recently emerged as one of the major interesting focuses. Recent studies have shown that GSK-3*β* is a vital factor in the inflammatory process [14–16]. GSK-3*β* inhibition has been successfully tested as a protective strategy in sepsis. Dugo et al. firstly demonstrated that the GSK-3*β* inhibition not only suppressed the production of proinflammatory cytokine but also increased the anti-inflammatory cytokine expression, and it provided a survival advantage and attenuated organ injury in LPS-induced sepsis models [16]. The protective effects of GSK-3*β* inhibition were further demonstrated in the live bacterial infection. Zhang et al. documented that the inhibition of GSK-3*β* suppressed the inflammatory responses and conferred a survival advantage in mice infected with* Francisella tularensis* [18]. Recently, using SB216763 as an inhibitor, Chang and colleagues found that GSK-3*β* inhibition reduced the proinflammatory cytokine expression and improved the survival rate in group A streptococcus-infected mice [17]. We previously demonstrated that GSK-3*β* inhibition by lithium reduced liver I/R injury, at least in part via inhibition of inflammation in rats [21]. In agreement with these studies, we demonstrated that inhibition of GSK-3*β* reduced inflammation, attenuated liver injury, and improved survival following CLP. These findings suggested that GSK-3*β* inhibition could protect liver against overwhelming inflammatory response during sepsis.

GSK-3*β* is known to regulate NF-*κ*B signaling, which plays a pivotal role in the pathophysiology of sepsis [25]. The transcription of proinflammatory mediators, including cytokines, chemokines, and NO, occurs mainly via the activation of NF-*κ*B. In patients with sepsis, circulating proinflammatory mediators were increased and correlated with the disease severity [26, 27]. Blockade of the activity of NF-*κ*B using genetic or pharmacologic approaches successfully reduced NF-*κ*B-mediated cytokines and inhibited multiple organ inflammation and injury during sepsis and/or endotoxemia, suggesting that inhibition of NF-*κ*B could be a promising therapeutic target in sepsis [28–30]. GSK-3*β* is able to affect NF-*κ*B activity by several different mechanisms. Dugo et al. demonstrated that GSK-3*β* inhibition reduced the Ser536 phosphorylation p65 but had no effect on the NF-*κ*B/DNA binding activity in the lung and reduced the increase in NF-*κ*B p65 activity caused by IL-1 in human embryonic kidney cells in vitro [16]. Martin et al. observed that GSK-3*β* regulated the production of proinflammatory and anti-inflammatory cytokines by LPS-stimulated monocytes via augmenting nuclear CREB activity and enhancing its association with CREB-binding protein while reducing the interactions between NF-*κ*B p65 and CBP [15]. In lithium-treated hepatocytes, Schwabe and Brenner documented that inhibition of GSK3 impaired NF-*κ*B activity, but I*κ*B degradation and NF-*κ*B DNA binding activity were not affected [31]. In this study, we identified GSK-3*β* regulated CLP-induced inflammatory cytokines production by differentially affecting the activation of NF-*κ*B and CREB.

There is increasing evidence that apoptosis is also involved in sepsis-induced liver dysfunction. Recent studies have demonstrated that GSK-3*β* inhibition protects renal cell from endotoxemia [19] and attenuates liver I/R injury [22] via inhibition of apoptosis. Our results demonstrated that GSK-3*β* inhibition inhibited apoptotic cell death. The mechanisms underlying the antiapoptotic effect of GSK-3*β* inhibition remain unclear. Apoptosis is potentially triggered by infiltrating neutrophil- or macrophage-derived cytokines such as TNF-*α*, ROS, and NO. Wang et al. observed that GSK-3 inhibitors downregulated LPS-induced renal cell apoptosis by reducing the amount of TNF-*α* production [19]. Moreover, results from studies of different types of liver damage also support the hypothesis that the protective effects of GSK-3*β* inhibition are via antiapoptosis. In a mouse model of liver I/R injury, Xia and colleagues showed that GSK-3*β* inhibitors increased *β*-catenin accumulation, which further enhanced antiapoptotic signaling through the induction of Bcl-2 and survivin expression [22]. This result was further supported by our previous study, which demonstrated that GSK-3*β* inhibition by lithium reduced hepatic apoptosis in liver I/R in rats [21]. Moreover, GSK-3*β* inhibition was shown to be capable of preventing apoptosis in D-galactosamine/LPS-induced liver injury by reducing endoplasmic reticulum stress [20].

## 5. Conclusion

In summary, we documented that GSK-3*β* inhibition could improve the survival of mice with polymicrobial sepsis, ameliorated liver injury, and reduced hepatic apoptosis. The mechanism appeared to involve its ability to inhibit inflammatory response via modulating NF-*κ*B and CREB activation. These findings would suggest that inhibition of GSK-3*β* may act as an alternative therapeutic strategy beyond antibiotic treatment.
